# Future of Poor-Law Infirmaries

**Published:** 1921-01-22

**Authors:** 


### Future of Poor-Law Infirmaries.

At the time when there is talk of not only closing hospitals, but of paying patients becoming quite general in our institutions, it is interesting to note what is happening in the Poor-law world—if the action of the Steyning Board of Guardians can be regarded as an index. They decided that, with a view of giving the nurses better facilities in regard to their training, a room, formerly used as a dispensary, be fitted up for use as an operating-theatre for minor operations, and that arrangements be made for the reception of paying patients, where minor operations only are required, and also for a maternity block. There was a good deal of opposition directed chiefly on the ground of expense—£8,000 to £10,000 were figures mentioned—and, despite assurances to the contrary that, as regards paying patients, the scheme would be self-supporting, and that practically no cost would be involved, the opposition were defeated by only two votes—13 to 11. The scheme also carried with it a request to the medical officer to consider the question of the administration of the infirmary as a separate institution apart from the workhouse. The charges to paying patients proposed under the scheme are as follow: Maintenance, nursing, etc., in surgical and medical cases, £2 2s. (minimum) to £3 3s. per week, according to means, the medical officer's fee for the operation and the services of the anæsthetist to be paid by the patient in addition; the charge for maintenance, nursing, etc., in maternity cases, £1 11s. 6d. (minimum) to £3 3s. per week, according to means, the medical officer's fee for attendance at the confinement to be paid by the patient in addition. And it may be added that the Steyning Union is one of the largest in the county of Sussex, and includes Brighton's wealthy sister town, Hove.

